# Characterization of subgroup J avian Leukosis virus isolated from Chinese indigenous chickens

**DOI:** 10.1186/s12985-018-0947-1

**Published:** 2018-02-13

**Authors:** Fanfeng Meng, Qiuchen Li, Yawen Zhang, Zhihui Zhang, Sibao Tian, Zhizhong Cui, Shuang Chang, Peng Zhao

**Affiliations:** 1 College of Veterinary Medicine, Shandong Agricultural University, Tai’an, Shandong China; 2 Shandong Provincial Key Laboratory of Animal Biotechnology and Disease Control and Prevention, Tai’an, Shandong China; 3 Shandong Provincial Engineering Technology Research Center of Animal Disease Control and Prevention, Tai’an, Shandong China

**Keywords:** Subgroup J, Avian leukosis virus, Chinese indigenous chickens, ENV, LTR, Chracterization

## Abstract

**Background:**

In spite of the purification of the laying hens and broilers of avian leukosis virus (ALV) has made remarkable achievements, the infection of ALV was still serious in Chinese indigenous chickens.

**Methods:**

In order to assess the epidemic state of avian leukosis virus in indigenous chickens in China, 10 novel strains of ALV subgroup J (ALV-J), named JS16JH01 to JS16JH10, were isolated and identified by virus isolation and immunofluorescence antibody assays from a Chinese local breed farm with a sporadic incidence of tumors. To understand their virological characteristics further, the proviral genome of *ENV-LTR* was sequenced and compared with the reference strains.

**Results:**

The homology of the *gp85* gene between the ten ALV-J strains and NX0101 was in the range from 89.7–94.8% at the nuclear acid level. In addition, their *gp85* genes were quite varied, with identities of 92–98% with themselves at the nuclear acid level. There were several snp and indel sites in the amino acid sequence of *gp85* genes after comparison with other reference strains of ALV. Interestingly, a novel insertion in the *gp85* region was found in two strains, JS16JH01 and JS16JH07, compared with NX0101 and HPRS-103.

**Discussion:**

At present, owing to the large-scale purification of ALV in China, laying hens and broiler chickens with ALV infection are rarely detected, but ALVs are still frequently detected in the local chickens, which suggests that more efforts should be applied to the purification of ALV from indigenous chickens.

## Background

Avian leukosis virus (ALV), belonging to the genus *Alpharetrovirus* of the family *Retroviridae*, is a notorious retrovirus that can cause various types of neoplastic diseases in birds [1]. ALV is divided into 10 different subgroups based on viral envelope interference, host range, and viral cross-neutralization patterns [2, 3]. Subgroups A, B, C, D, J, and K are exogenous viruses that infect chickens [3, 4], whereas subgroup E is an endogenous virus [5]. Avian leukosis is a neoplastic disease that primarily causes myelocytomatosis (ML) and physiological hemangioma [3, 6, 7]. ALV-J was first isolated and identified from commercial meat-type chickens in 1988 in the United Kingdom [8], and then it spread quickly worldwide causing enormous economic losses to the global poultry industry [6, 9–13]. In 1999, ALV-J infection was first detected in broilers in China [14] and subsequently spread to other chicken types. Chickens infected with ALV-J have been a serious issue in the China poultry industry since 2007 [15, 16].

A measure of success has been achieved in the purification, prevention, and control of ALV among white-feathered broilers and layer chickens since the purification of ALV was executed in China. But there are many breeds with excellent characteristics in China, these chickens are still susceptible to ALV-J infection [17, 18].Thus, to grasp the quasispecies evolution of ALV in a flock of chickens of local breed and accelerate the purification of ALV in China, this study isolated and identified the viruses from a local chicken farm during the process of purifying ALV and the molecular characteristics of *env* and *LTR* were determined and analyzed.

## Methods

### Case history

In an indigenous chicken farm, chickens suffering from hepatosplenomegaly, yellowish-white tumors on the visceral surface, and presenting a drastic decline in egg production were suspected to be infected with ALV. Necropsies were performed on the diseased birds soon after death. Part of the tissues with altered local pathology were fixed with 4% formalin for pathological section.

### Virus isolation and identification

Thirty chickens were randomly selected and plasma samples were collected in sterile 2-mL tubes containing 1% sodium heparin. After centrifugation at 2000 rpm for 3 min, each plasma sample was inoculated into DF-1 cells originating from embryonic fibroblast cells of C/E type chickens resistant to subgroup E endogenous ALV. The cultures were maintained for 9 days in Dulbecco’s modified Eagle’s medium supplemented with 2% fetal bovine serum at 37 °C in a 5% CO2 incubator. Next, each supernatant sample was subjected to ELISA using an ALV antigen test kit (IDEXX, USA) to detect the ALV group-specific antigen p27. Meanwhile, the cultured cells were fixed by using an aldehyde fixative and then were detected by immunofluorescence antibody assays for ALV-J, ALV-A/B, reticuloendotheliosis (REV), and Marek’s disease virus (MDV) infection by using ALV-A/B specific polyclonal antibody, ALV-J specific monoclonal antibody JE9, REV specific monoclonal antibody 11B118, and MDV specific monoclonal antibodies BA4 and H19, respectively. Then FITC-labeled anti-mouse IgG secondary antibody was used to detect primary antibody binding, after that the cells were observed by using a fluorescence microscope.

### Cloning and sequencing of *ENV*

A pair of primers (ALV-F: GATGAGGCGAGCCCTCTCTTTG; ALV-R: TGTGGTGGGAGGTAAAATGGCGT) used to target the entire sequence of env was designed to specifically amplify a highly conserved region present in all ALV subgroups. PCR was performed as described by Meng et al. [19] with the DNA of p27-positive DF-1 cells. PCR products were separated by 1.0% agarose gel electrophoresis, purified by using the Omega Gel Extraction kit (Omega, USA),and were then cloned into the PMD18-T vector. The resulting construct was then used to transform *Escherichia coli* DH5α cells (TaKaRa, Japan). The positive clones were then sequenced by Shanghai Sangon Biotech Co., Ltd. (Shanghai, China).

### Analysis of ALV-J strains and molecular evolution

The homologies of the nucleic acid and amino acid sequences were compared between gp85 from each ALV-J strains and the reference sequences. To assess the trends in molecular evolution of the ALV-J strains, gp85 from different ALV reference strains were included for phylogenetic analysis (Table 1). Phylogenetic analysis was accomplished by muscle alignment and the neighbor-joining and maximum parsimony methods with 500 bootstrap replicates using software MEGA 5.1.

**Table 1:** Reference ALV strains for the comparison of *gp85* amino acid sequences

Strain	Year	Location	Genbank
A-MAV	1993	–	L10922
A-RSA	1990	France	M37980
B-MAV	1993	–	L10924
B-RSR	1998	USA	AF052428
C-Prague	1982	USA	J02342
D-RSR	1992	Japan	D10652
Ev-3	1991	–	M60397
SD0501	2007	Shandong	EF467236
JS11C1	2013	Jiangsu	KF746200
JS14CZ01	2017	Jiangsu	KY490695
HPRS103	2006	USA	EF058157
NX0101	2005	China	DQ115805
BJ0301	2003	Beijing	AY897230
CAUSY01	2009	Beijing	HM640945
HB0301	2003	Hebei	AY897229
HN1001–1	2001	Henan	HQ260974
HNN	2009	Unknown	HM235668
JL08CH3–1	2008	Jilin	HQ634809
SCDY1	2010	Sichuan	HQ425636
SD0201	2002	Shandong	AY897218
SD0301	2003	Shandong	AY897228
SD100502J	2010	Shandong	HQ270188
WM100402	2010	Anhui	HQ271448
SD13QJ03	2015	Shandong	KM873194
JS13LY02	2014	Jiangsu	KM873181
JS13LH03	2014	Jiangsu	KM873180
JS13DX06	2014	Jiangsu	KM873178
JS13TH01	2014	Jiangsu	KM873184
JS14XJ01	2014	Jiangsu	KM873185
SD12HN04	2014	Shandong	KW873190

## Results

### Clinical and histopathological analysis

**Fig. 1:**
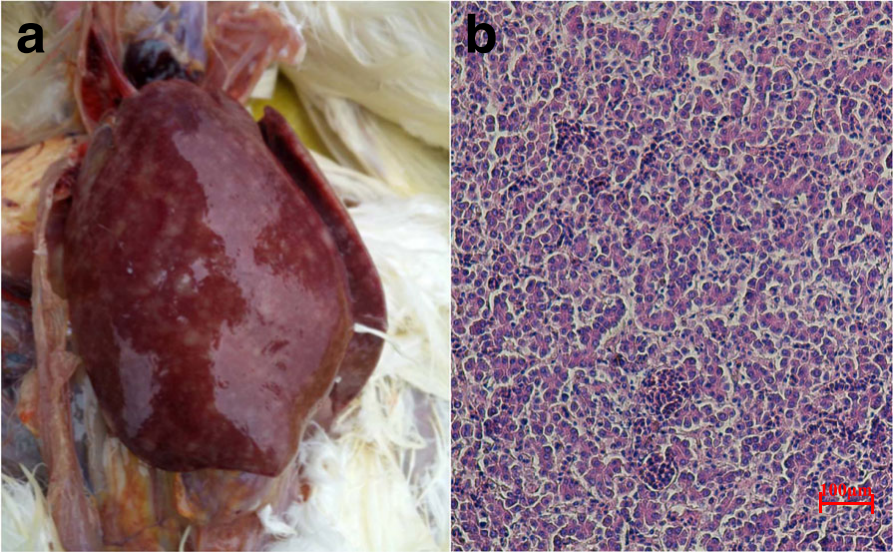
Gross lesions and histological appearance of the sick chickens. **a** liver enlarged with tumor; **b** eosinophilic myeloid cells hyperplasia in liver

Necropsies were performed on the diseased birds soon after death. Hepatosplenomegaly and yellowish-white tumors on the visceral surface were the apparent symptoms (Fig. 1a). Histolopathological analysis of the liver revealed infiltration of eosinophilic myeloid cells around the hepatocytes (Fig. 1b).

### Isolation and identification of exogenous ALV

**Fig. 2:**
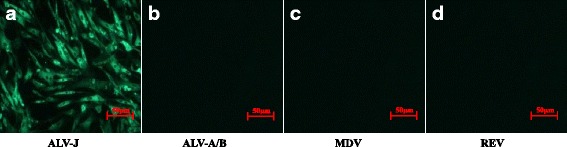
Detection of ALV-J, ALV-A/B, MDV and REV in DF-1 cells by immunofluorescence assay **a** The result of IFA mediated with ALV-J-specific monoclonal antibody JE9; **b-d** The IFA results for ALV-A/B; **c** The IFA results for MDV; **d** The IFA results for REV

Ten strains of ALV-J, named as JS16JH01–JS16JH10 were isolated from an indigenous chicken farm in China. The ALV p27 antigen was detected in the supernatants of the DF-1 and CEF cultures inoculated with the plasma of isolated strains. The results showed that 10 samples inoculated with plasma samples were positive for ALV p27 antigen, whereas the samples of the control group were negative, and it indicated that the chickens were infected with exogenous ALV. Immunofluorescence antibody assays revealed that the infected cells were only labeled with ALV-J specific monoclonal antibody JE9; no reaction with ALV-A/B specific polyclonal antibodies, REV specific and MDV specific monoclonal antibodies (Fig. 2) was shown.

### Sequence analysis of the *ENV* and *LTR* gene

**Fig. 3:**
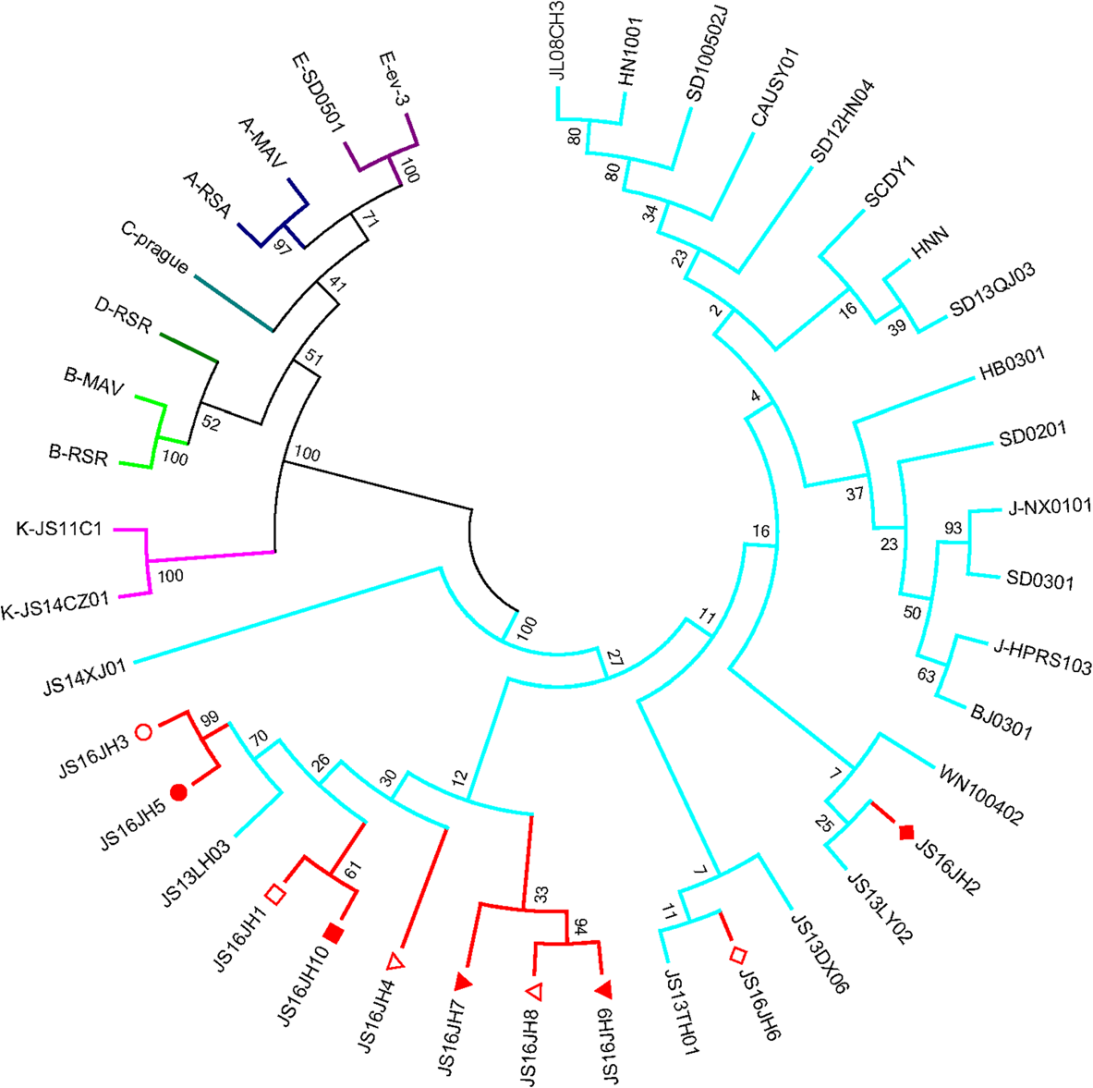
Phylogenetic analysis for gp85 sequences of the isolated strains and other ALV reference strains of different subgroups. Numbers at the branch points in the tree are bootstrap values and the isolated strains are indicated with different shapes

Target fragments of approximately 2200 bp were amplified from the DF1 cells infected with ALV-J using the common primers (Genbank accession no. MG700533-MG700542). Then gp85 sequences were obtained, and were compared with the sequences of reference strains of different subgroups. The results showed that the gene homology with ALV-J ranged from 89.7–94.8%, but no more than 51.8% compared with the other subgroups. What is more, their gp85 genes were quite varied, with identities of 92–98% with themselves at the nuclear acid level. Furthermore, phylogenetic analysis revealed that all the 10 strains were on the same branch as the ALV-J reference sequence (Fig. 3).

**Fig. 4:**
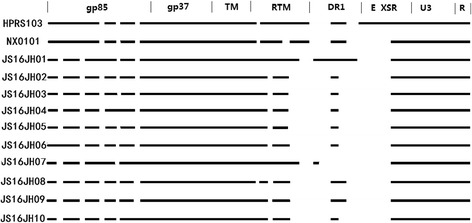
The atlas of *env + LTR* gene of the 10 isolated strains and reference strains. The blank in the straight line means the missing sites in the strain

Further analysis suggested that there were multiple deletions and mutations in the *gp85* gene in the isolated strains in comparison with NX0101 and HPRS-103 (Fig. 4). Compared with NX0101, 60 nucleotides were missed in *rTM* and DR-1. In addition, a missing asparagine and a missing arginine in the 62nd and 119th amino acid positions were observed. Furthermore, A143G, R151P, and P154S, from most of the isolated strains, changed the hydrophilicity of the amino acid in the cells and the advanced structure, as well as the function, of the protein (Table 2).

**Table 2:**
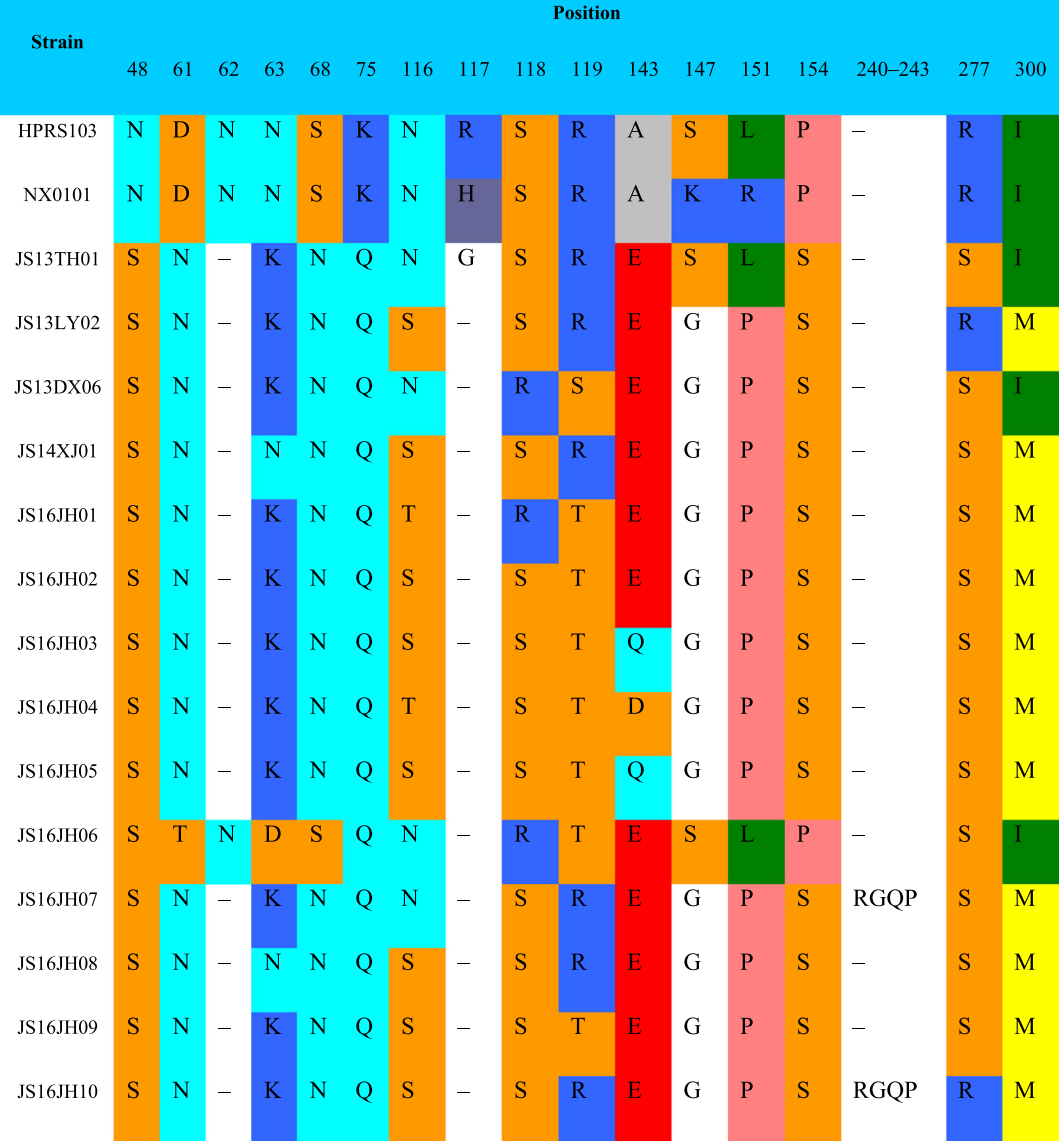
Deletion and mutations in gp85 protein of the isolated strains

Note: The different capital letters in the table indicate the abbreviations of amino acids in each position, “-” means the deficiency of amino acid in this site. Each amino acid has a specific color according to Shapely model

## Discussion

ALV-J was first detected in China in 1999 [14], which was then followed by more reports on white-feathered broilers, layer chickens, and local breed chickens infected with ALV-J [20–22]. From 2007 to 2010, ALV-J caused an outbreak of hemangioma, which led to enormous economic losses to the Chinese poultry industry [15, 16]. Owing to China’s 11th Five-Year plan, high achievement was made in the purification process of avian leukosis from chicken farms; there has been fewer reports related to ALV-J in laying hens and broiler [23]. However, the strong pathogenicity and diversity of ALV still render indigenous chickens susceptible [24, 25] and seriously affect the production and promotion of excellent chicken varieties in China. In a previous national epidemiological survey, a significant proportion of local breeds (for example, 70–80%) were found to be infected with ALV-J. All these available data indicated that indigenous breeds in China were greatly infected with ALV-J.

Indigenous chickens infected with myeloma leukemia caused by ALV-J were first detected in the Shandong province in 2005; a death rate of about 10% occurred on the farm [26]. Lin [27] isolated 12 strains of avian leukosis virus subgroup J from chickens in south China during 2013–2014 and detected several amino acid variations and potential glycosylation sites in gp85 by sequencing. In addition, they found that many genetic variations of the gp85 exist in an indigenous chicken flock [17]. The monitoring of ALV in local chickens in our country has been continuous; simultaneous, our laboratory has executed the purification of ALV-J from indigenous chickens.

In this study, 10 ALV strains were isolated from golden silky fowl by a quality gene bank analysis during the process of purification. By virus isolation and identification, sequencing, and phylogenetic analysis, it was found they all belonged to subgroup J. On the phylogenetic evolutional tree, all the 10 strains were placed on the same branch as the ALV-J referential sequences. Gp85 nucleic acid homology ranged from 89.6–92.0% compared with NX0101 whereas the homology was between 84.3% and 87.3% at the amino acid level. However, gp85 sequences of the strains were quite disparate, with identities of only 50% compared with those of other subgroups. Owing to the high error and high recombination rates of polymerase, RNA viruses are highly susceptible to genetic variation and ALV-J also exhibits extremely high mutation levels. Gp85 homology between the strains was 86.1–98.4% at the nucleic acid level and 86.1%–98.4% at the amino acid level. These data indicate that an enormous diversity of ALV exists among indigenous chickens.

The envelope protein gp85 plays an essential role in the viral neutralization response and specificity of virus identifying host cells. Gp85 genes of the strains isolated in the present study showed several amino acid deletions and mutation sites compared with the reference strains. Asparagine and arginine residues were missing at the 62nd and 119th positions, respectively. Most of the mutations (e.g., A143G, R151P, and P154S) in the isolated strains, when compared with the reference strains, changed the hydrophilicity of the amino acid in the cells, and the higher structure or the function of protein. All changes affected the identities of virus and caused the virus to evolve and form a new branch.

## Conclusions

The 10 strains of ALV-J isolated from the indigenous chickens in this study deviated from the original Chinese strains, suggesting that chickens in China may be a gene pool for ALV and the presence of large numbers of variants in chickens of local breeds. Moreover, our results indicated the rapid evolution of ALV-J during the process of purification. This study focused on the characterization of ALV-J and has helped us to further understand the diversity and complexity of ALV in the local chickens; importantly, it has informed us that more efforts should be put towards the purification of indigenous chickens.
